# The combined utilization of Chlorhexidine and Voriconazole or Natamycin to combat *Fusarium* infections

**DOI:** 10.1186/s12866-020-01960-y

**Published:** 2020-09-05

**Authors:** Tao Jiang, Jing Tang, Zhiqin Wu, Yi Sun, Jingwen Tan, Lianjuan Yang

**Affiliations:** 1 Department of Clinical Laboratory, Jingzhou Central Hospital, The Second Clinical Medical College, Yangtze University, Jingzhou, 434100 China; 2 Department of Stomatology, Jingzhou Central Hospital, The Second Clinical Medical College, Yangtze University, Jingzhou, 434100 China; 3 Department of Ophthalmology, Jingzhou Central Hospital, The Second Clinical Medical College, Yangtze University, Jingzhou, 434100 China; 4 Department of Dermatology, Jingzhou Central Hospital, The Second Clinical Medical College, Yangtze University, Jingzhou, 434100 China; 5 Department of Medical Mycology, Shanghai Skin Disease Hospital, Tongji University School of Medicine, Shanghai, 200443 China

**Keywords:** *Fusarium*, Chlorhexidine, Voriconazole, Natamycin, Synergistic

## Abstract

**Background:**

*Fusarium* species are the fungal pathogens most commonly responsible for the mycotic keratitis, which are resistant to the majority of currently available antifungal agents. The present study was designed to assess the efficacy of a combination of low doses chlorhexidine with two other commonly used drugs (voriconazole and natamycin) to treat *Fusarium* infections.

**Results:**

We utilized combinations of chlorhexidine and natamycin or voriconazole against 20 clinical *Fusarium* strains in vitro using a checkerboard-based microdilution strategy. In order to more fully understand the synergistic interactions between voriconazole and chlorhexidine, we utilized a *Galleria mellonella* model to confirm the combined antifungal efficacy of chlorhexidine and voriconazole in vivo. We found that for voriconazole, natamycin, and chlorhexidine as single agents, the minimum inhibitory concentration (MIC) ranges were 2–8, 4–16, and > 16 μg/ml, respectively. In contrast, the MIC values for voriconazole and chlorhexidine were reduced to 0.25–1 and 1–2 μg/ml, respectively, when these agents were administered in combination, with synergy being observed for 90% of tested *Fusarium* strains. Combined chlorhexidine and natamycin treatment, in contrast, exhibited synergistic activity for only 10% of tested *Fusarium* strains. We observed no evidence of antagonism. Our in vivo model results further confirmed the synergistic antifungal activity of chlorhexidine and voriconazole.

**Conclusions:**

Our results offer novel evidence that voriconazole and chlorhexidine exhibit synergistic activity when used to suppress the growth of *Fusarium* spp., and these agents may thus offer value as a combination topical antifungal treatment strategy.

## Background

Keratomycosis is a form of fungal infection that can be challenging to treat, and that can result in permanent and severe vision damage when not adequately treated [1]. *Fusarium* species are the causative pathogens in between 36 and 67% of all traumatic keratitis cases, in which *F. oxysporum* was the most frequently isolated species followed by *F. solani* [2]. *Fusarium*-related keratitis remains challenging to treat as these fungi are intrinsically resistant to most available antifungal agents. While advances in the standard treatment for keratitis have been developed in recent years, with natamycin (NAT) and voriconazole (VOR) being the current treatment agents of choice, further optimization of these therapeutic regimens is still warranted [3].

While previous studies have shown that NAT can be effective for the treatment of *Fusarium* infections, and 5% NAT is currently the first-line treatment for mycotic keratitis in certain nations, the poor penetration of this compound has been linked to failed treatment in some cases [1]. More recently, the application of 1% topical VOR has been shown to be an effective means of treating refractory fungal keratitis [4] while also exhibiting satisfactory diffusion within the aqueous humor. However, single-agent VOR treatment has not been shown to be adequately protective as a means of treating some patients, suggesting that combination therapies may be necessary to achieve reliable and durable therapeutic efficacy [5]. Chlorhexidine (CHL) is an antiseptic agent that is commonly used and has been shown to be safe for ophthalmic exposure at concentrations of 1% or below [6]. Furthermore, the intravitreal injection of 0.1% CHL has been shown to be a safe antiseptic strategy [7]. The utility of CHL for treating keratomycosis, however, remains to be established.

The goal of the present study was to assess the impact of combinations of NAT or VOR with CHL on clinical *Fusarium* isolates. We employed a checkerboard microdilution strategy to reliably identify potentially useful combinations of these therapeutic agents in vitro. We then employed a *G. mellonella* model to validate our findings in vivo.

## Results

### Assessment of the in vitro antifungal activity of CHL, NAT, and VOR

CHL, VOR, and NAT solutions exhibited Minimum inhibitory concentration (MIC) values of > 16 μg/ml, 2–8 μg/ml, and 4–16 μg/ml to the *Fusarium* isolates, respectively. CHL did not exhibit antifungal activity, even at the highest tested concentration. When combined with VOR, the MIC values for CHL and VOR were reduced to 1–2 μg/ml and 0.25–1 μg/ml, respectively, with synergy being observed for 18 *Fusarium* strains (90%). In contrast, such synergistic interactions were only observed for 2 *Fusarium* strains (10%) treated with a combination of CHL and NAT (Table. 1). We did not observe any evidence of antagonism in any of these analyses.

**Table 1:** Combination activity of CHL with VOR or NAT against *Fusarium* species

Strains	Origin	MICs (μg/ml)				MICs (μg/ml)		
		CHL	VOR	CHL/VOR	FICI	NAT	CHL/NAT	FICI
***F. Solani***								
Jzfs1	Skin	> 16	4	1/0.5	SYN	8	2/8	N
Jzfs2	Skin	> 16	2	1/0.5	SYN	8	2/8	N
Jzfs3	Cornea	> 16	2	1/1	N	8	1/8	N
Jzfs4	Cornea	> 16	8	2/0.5	SYN	4	2/4	N
Jzfs5	Skin	> 16	2	2/0.5	SYN	8	2/8	N
Jzfs6	Cornea	> 16	4	1/0.5	SYN	16	2/4	SYN
Jzfs7	Skin	> 16	4	1/0.5	SYN	8	1/4	N
Jzfs8	Auditory canal	> 16	2	1/0.5	SYN	8	1/4	N
Jzfs9	Auditory canal	> 16	2	1/0.5	SYN	8	1/4	N
Jzfs10	Nail	> 16	2	2/0.5	SYN	4	2/4	N
Jzfs11	Nail	> 16	2	1/0.5	SYN	8	1/4	N
Jzfs12	Cornea	> 16	4	1/0.5	SYN	8	1/4	N
***F. oxysporum***								
Jzfo1	Cornea	> 16	2	1/0.25	SYN	8	2/4	N
Jzfo2	Nail	> 16	4	2/0.5	SYN	8	1/8	N
Jzfo3	Skin	> 16	4	2/0.5	SYN	8	1/2	SYN
Jzfo4	Skin	> 16	2	1/1	N	8	2/8	N
Jzfo5	Cornea	> 16	2	1/0.25	SYN	8	2/4	N
Jzfo6	Nail	> 16	2	1/0.5	SYN	4	1/4	N
Jzfo7	Auditory canal	> 16	2	1/0.5	SYN	8	1/4	N
Jzfo8	Nail	> 16	2	1/0.5	SYN	8	1/4	N

*SYN* synergy (FICI ≤0.5), *N* indifference (no interaction 0.5 < FICI≤4), *CHL* Chlorhexidine, *VOR* Voriconazole, *NAT* Natamycin, *MIC* minimum inhibitory concentration, *FICI* Fractional inhibitory concentration index

### Assessment of the in vivo antifungal activity of CHL and VOR in *G. mellonella*

**Fig. 1:**
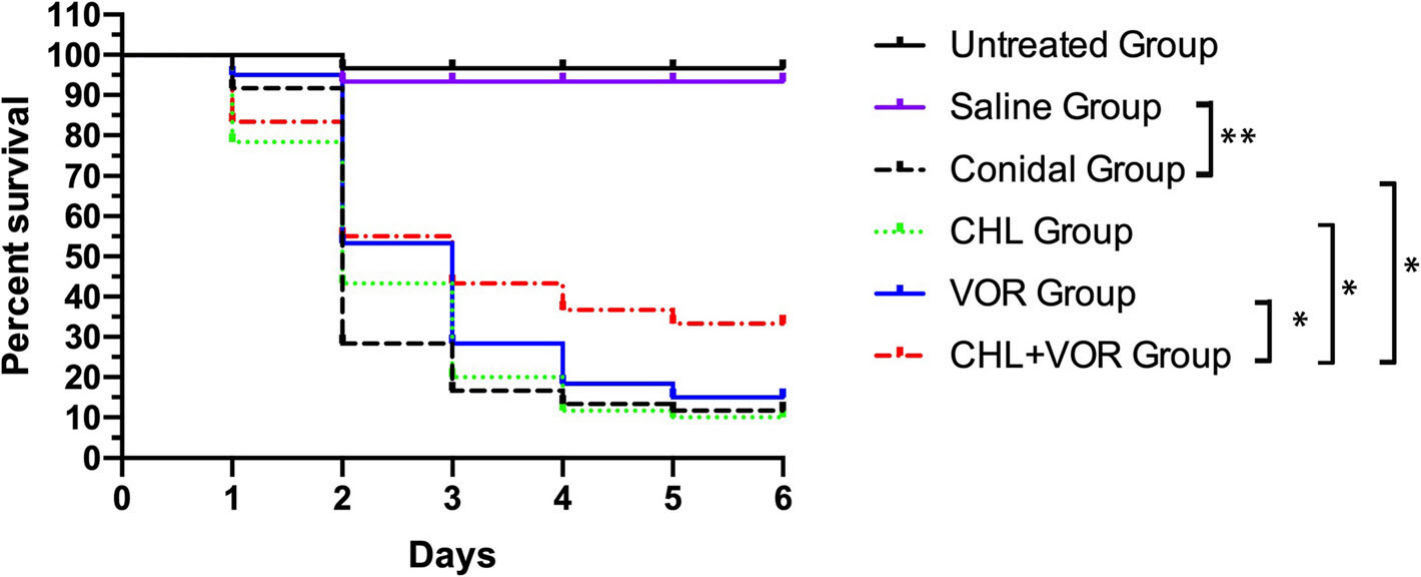
*G. mellonella* survival rates. Untreated Group, wild type lava without *Fusarium* infection; Saline Group, wild type larvae injection with saline; Conidial Group, larvae infection with *Fusarium* without any treatment; CHL Group, *Fusarium* infected larvae treated with CHL only; VOR Group, *Fusarium* infected larvae treated with VOR only; CHL + VOR Group, *Fusarium* infected larvae treated with CHL combined with VOR; CHL: Chlorhexidine; VOR: Voriconazole; NAT: Natamycin. The experiment was repeated thrice on different days. *, *p* < 0.05; ** *p* < 0.0001

In order to evaluate the synergistic efficacy of CHL and VOR in vivo*,* we infected *G. mellonella* with *F. solani* Jsfs1 and then treated these larvae using CHL and/or VOR. The survival in groups treated with VOR, CHL, and VOR with CHL was 15%, 10% and 33.3%, respectively. VOR treatment slightly improved larval survival, whereas CHL alone failed to improve larval survival, compared with the conidia group. Treatments with VOR combined with CHL significantly (*P <* 0.05) prolonged the survival of larvae (Fig. 1). Together, these in vivo findings thus confirmed the synergistic antifungal activity of CHL and VOR as evidenced by improved larval survival.

### Hi**s**topathological analyses

**Fig. 2:**
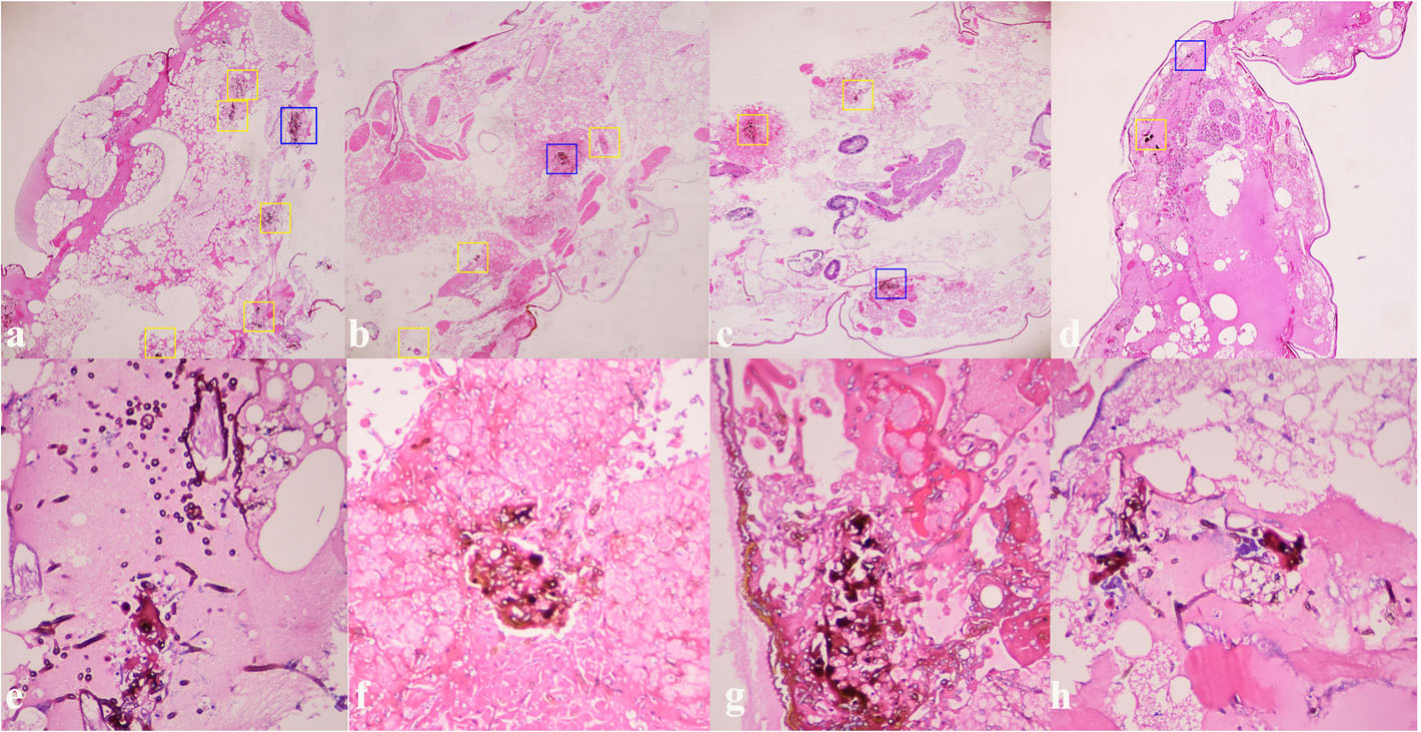
*G. mellonella* histopathology. **a**-**d**, HE, 100×; **e**-**h**, HE, 400×. **a** and **e**, Saline treatment group; **b** and **f**, CHL treatment group; **c** and **g**, VOR treatment group; **d** and **h**, CHL combine VOR treatment group; Yellow and blue frame, *F. solani* spore and hyphae clusters; Blue frame, The magnification part

On day 3 post-*F. solani* Jzfs1 infection, we conducted a histopathological assessment of larvae in this study. We observed the formation of *F. solani* spore and hyphae clusters in infected tissues regardless of treatment status (Fig. 2). There were 6, 4, 3 and 2 visible fungal clusters in the control group (Fig. 2a and e), CHL group (Fig. 2b and f), VOR group (Fig. 2c and g) and CHL + VOR group (Fig. 2d and h), respectively. VOR treatment was associated with a slight reduction of the number of visible fungal clusters relative to control and CHL groups. The combination treatment group exhibited dramatic reductions of the number of visible fungal clusters relative to the other three evaluated groups.

## Discussion

Herein we evaluated the potential combination antifungal activity of antifungal agents against pathogenic *Fusarium* spp. CHL, which is a common, inexpensive, safe, and efficacious antiseptic agent exhibited promising performance. CHL is functions by binding to cell membranes and thereby impairing bacterial adhesion and driving the leakage of bacterial cellular contents [8]. Most bacterial and *Candida* species have been shown to be killed by 1–2% CHL solutions [9], while CHL concentrations of 1% or lower have been shown to be safe when used for ophthalmic purposes [10]. There are several studies showing that CHL exhibits in vitro antifungal activity against *Fusarium* spp. Oliveira et al. found that CHL exhibited fungicidal activity against 90% of tested *F. oxysporum* strains and 100% of tested *F. solani* strains when evaluating 98 strains isolated from fungal keratitis patients [11]. Xu et al. found that the MIC range for CHL is 8–32 μg/mL, while the MIC_90_ value of chlorhexidine was 32 μg/mL for 24 *F. solani* strains [12]. In the Netherlands between 2005 and 2016, 89 cases of *Fusarium* keratitis from 16 different hospitals were identified, and in vitro susceptibility testing indicated that chlorhexidine was active against *Fusarium* spp. with a MIC range of 8–32 mg/L for *F. solani* and 1–64 mg/L for *F. oxysporum* [5]. In our study, we found that CHL did not cause any detectable inhibition of *Fusarium* species when used as a single agent at the highest tested concentration (MIC > 16 μg/mL). The difference between our findings and these prior studies may be attributable to the fact that relatively few fungal isolates were tested and that lower concentrations of CHL were employed herein.

In two blinded randomized trials by the same investigators, they observed patients of fungal keratitis treated with natamycin compared to CHL gluconate at various concentrations. Their results indicated that 0.2% CHL yielded the best results [13, 14]. However, the overall estimate of effect was uncertain [15]. Fungal keratitis caused by *F. solani* has been successfully treated with a combination of 0.02% CHL and AMB (Amphotericin B), underscoring the potential of CHL as an approach to the clinical management of fungal keratitis [16]. However, data regarding treatment with a combination of CHL and VOR is still limited.

In this study, we evaluated therapeutic interactions between CHL and VOR or NAT via a checkerboard microdilution strategy. As a first-line drug used for the management of fungal keratitis, NAT exhibited poor synergy ability with CHL both in vitro and in vivo. In contrast, the combination of CHL and VOR treatment exhibited synergistic activity against 90% of tested *Fusarium* strains. We observed no evidence of antagonism. As *G. mellonella* exhibit immunological response similar to those of mammals and are easy to manipulate, can be maintained at low costs, and incur minimal ethical concerns [17], they can be used as an ideal model system for studies of fungal virulence and antifungal drug activity [18]. We therefore utilized a *G. mellonella* model to evaluate the in vivo synergistic activity of these treatments. We determined that combination VOR + CHL treatment of infected *G. mellonella* larvae was associated with significant increases in larval survival. What’s more, the concentration of CHL is about 0.00015%, which is very low and proved to be safe when used in ophthalmic [10]. The mechanisms underlying this synergy are likely attributable to the ability of CHL to increase VOR penetration and/or to direct damage to *Fusarium* cell membranes without affecting drug efflux pump activity [19].

In order to expand upon these in vivo studies, we additionally conducted microscopic analyses of infected larvae (Fig. 2), which confirmed that combination treatment was associated with a reduction in the degree of tissue damage observed in *G. mellonella* larvae relative to control groups, suggesting that these two compounds exhibit excellent synergy in vitro and in vivo and are thus ideal for treating *Fusarium* keratitis.

## Conclusions

Our results demonstrate that CHL and VOR exhibit synergistic efficacy against *Fusarium* species in vitro and in vivo*.* These findings suggest that CHL and VOR may be a viable therapeutic combination treatment for *Fusarium* infections, although future clinical trials and studies will be needed to validate this finding and to explore the underlying molecular mechanisms.

## Methods

### Fungal strains

In total, we obtained 20 clinical *Fusarium* isolates (12 *F. solani* and 8 *F. oxysporum* strains) from clinical cultures (Table 1). These fungi were identified based upon a combination of morphological analyses and sequencing of the internal transcribed spacer (ITS) rDNA and translation elongation factor (TEF) 1α coding regions [20].

### Antifungal agents

VOR (purity≥99%), NAT (purity≥99%) and CHL (purity≥99%) were obtained as powders from Selleck Chemicals (TX, USA), and were dissolved with DMSO (Amresco, OH, USA) to prepare 1600 μg/mL stock solutions.

### Inoculum preparation

*Fusarium* strains were grown for 7 days at 30 °C on Sabouraud dextrose agar (SDA), after which they were isolated and resuspended in a 2 mL volume of sterile saline. Sterile gauze was utilized to filter conidia suspensions, after which a hemocytometer was used to quantify concentrations therein, which were adjusted to 1–5 × 10^6^ cfu/mL.

### Assessment of single-agent antifungal activity in vitro

We conducted antifungal susceptibility tests based upon the CLSI M38-A2 [21]. Briefly, stock solutions were diluted in a two-fold serial manner using RPMI-1640 (Gibco, NY, USA) to yield final concentrations of 0.0313–16 μg/ml. Microdilution wells were then filled with 100 μL of appropriate *Fusarium* isolates at 1–5 × 10^4^ cfu/mL. Plates were incubated for 48 h at 35 °C, after which MIC values were determined by identifying the minimum antifungal agent dose necessary to achieve 100% inhibition of fungal growth relative to control untreated wells. We additionally included *A. flavus* strain ATCC 204304 as a quality control strain in the present analysis.

### Assessment of in vitro interactions between CHL and VOR or NAT

A checkerboard microdilution strategy was used to evaluate synergistic interactions between CHL and NAT or VOR against *Fusarium* strains, with this approach having been adapted from the CLSI M38-A2 microdilution method. Briefly, we added 50 μl volumes of serially-diluted VOR or NAT horizontally across microdilution plates, while 50 μl CHL samples that had been serially diluted were added in a vertical direction, with 100 μl of a prepared inoculum suspension also being added to each well. Incubation times and MIC determinations for this assay were as above. Combination drug interactions were classified based upon the fractional inhibitory concentration index (FICI) [22] which was calculated as follows: FICI = (MIC A in combination/MIC A alone) + (MIC B in combination/MIC B alone). Synergy was said to exist if FICI was ≤0.5, while the interaction was said to be indifferent when FICI was > 0.5 and < 4.0, and antagonistic when FICI was ≥4.0. Assays were conducted in duplicate on different days to ensure validity.

### *G. mellonella* survival assays

*Galleria mellonella* caterpillars from the final instar larval developmental stage (Chengdu Pets and Insects Company, Sichuan, China) were maintained under dark conditions and were utilized within 1 week of receipt. In total, 20 randomly selected larvae (330 ± 25 mg, 2–3 cm) were utilized per group. Two control groups were injected with 10 μL of saline or with no solution, respectively. Infected animals were injected with a 10 μl volume containing *Fusarium* Jzfs1 (1 × 10^7^/mL) using a 25 μl Hamilton syringe. Injections were made into the hemocoel of each larva through the last left proleg, with this region having first been cleaned with an alcohol swab. Following the completion of this injection process, larvae were transferred to plastic containers at 37 °C, with survival being assessed each day over a 6-day period. All experiments were conducted in triplicate.

### Assessment of VOR and CHL efficacy for the treatment of *Fusarium* infections alone or combination in *G. mellonella*

*Galleria mellonella* killing assays were conducted at 37 °C, as above, with 1 × 10^7^/ml cells/larva being used for initial inoculation. VOR and CHL were diluted in saline and then used to treat infected *G. mellonella* in the following combinations: VOR (3 μg/mL), CHL (1.5 μg/mL), and VOR + CHL (3 μg/mL and 1.5 μg/mL, respectively). As controls, additional *G. mellonella* caterpillars were injected twice using saline. All drugs were injected through the last left proleg of each larva. The *G. mellonella* survival curves were analyzed by the Kaplan-Meier method, and differences were determined by using the log-rank (Mantel Cox) test. Differences were considered significant at *P* values of < 0.05.

### Histological analyses

*Fusarium* presence within *G. mellonella* tissues was assessed via collecting larvae from each group on day 3 post-infection and treatment. These larvae were fixed with 10% neutral formalin, after which they were dehydrated with an ethanol gradient (70, 80, 90, 96, and 100% ethanol). Samples were then paraffin and xylene embedded, sliced to prepare 8 μm sections, and stained with hematoxylin and eosin (HE). Samples were then evaluated via an FSX100 fluorescence microscope (Olympus, Tokyo, Japan) at 10× and 40×. As controls, saline-injected larvae were also collected.

## Data Availability

All data generated or analyzed during this study are included in this published article. Access to raw data can be acquired by connecting to the corresponding author via email.

## Abbreviations

MIC: Minimum inhibitory concentration
FICI: Fractional inhibitory concentration index
NAT: Natamycin
VOR: Voriconazole
CHL: Chlorhexidine
AMB: Amphotericin B
ITS: Internal transcribed spacer
TEF: Translation elongation factor
